# Comparison of Male and Female Sexual Dysfunction between Hemodialysis and Peritoneal Dialysis in Patients with End-Stage Renal Disease: An Analytical Cross-Sectional Study

**DOI:** 10.1155/2022/9404025

**Published:** 2022-04-18

**Authors:** Shahryar Zeighami, Saeid Dehghankhalili, Khadije Heiran, Seyede Pegah Azarchehry, Alireza Heiran, Mehrab Sayadi, Fatemeh Azadian

**Affiliations:** ^1^Department of Urology, Shiraz University of Medical Sciences, Shiraz, Iran; ^2^Student Research Committee, Shiraz University of Medical Sciences, Shiraz, Iran; ^3^Department of Midwifery, School of Nursing and Midwifery, Zahedan University of Medical Sciences, Zahedan, Iran; ^4^Department of Biochemistry, Faculty of Biological Sciences, Tarbiat Modares University, Tehran, Iran; ^5^Non-Communicable Diseases Research Center, Shiraz University of Medical Sciences, Shiraz, Iran; ^6^Cardiovascular Research Center, Mohammad Rasoolallah Research Tower, Shiraz University of Medical Sciences, Shiraz, Iran

## Abstract

**Background:**

Maintenance dialysis is the most common treatment for end-stage renal disease (ESRD) patients. One of the most ignored but important health issue among dialysis patients is sexual dysfunction, which interferes with quality of life (QoL). Studies showed that the side effects of the two conventional methods of dialysis (hemodialysis (HD) and peritoneal dialysis (PD)) are different on a patient's health. Therefore, we sought to compare the sexual dysfunction score, both male and female, between patients undergoing HD and PD.

**Methods:**

One hundred seventy adults (85 HD and 85 PD) subject with end-stage renal disease (ESRD) on dialysis for at least 2 months were included. For male subjects, the erectile function (EF) domain of the International Index of Erectile Function (IIEF) questionnaire was calculated. Moreover, the Female Sexual Function Index (FSFI) questionnaire was calculated for females. Data were analyzed via SPSS software. Two independent sample *t*-test with two-sided significance level of 5% was used for comparing the sexual dysfunction score between HD and PD patients.

**Results:**

Out of 170 patients with mean age of 49.34 ± 11.7 years, 52.9% were female. Better sexual function scores were obtained in the HD group's females for desire, orgasm, and satisfaction domains, as well as the total score (*P* = 0.03, 0.016, 0.02, and 0.039, respectively). The erectile function was significantly better in the PD group's males (*P*).

**Conclusion:**

We found better sexual life in the HD group's females and PD group's males. Considering dialysis as a life-long treatment of CKD patients, this part of a patient's life must be taken seriously by the healthcare providers to choose the most suitable method for patients based on their personalized conditions.

## 1. Introduction

Chronic kidney disease (CKD) is projected to increase all over the world. The global prevalence of CKD reported in different studies is heterogeneous. Based on a systematic review in 2016, the global prevalence of CKD was 13.4%. CKD inevitably leads to end-stage renal disease (ESRD), which has a high mortality rate [1]. Dialysis is the most common treatment for ESRD patients who cannot undergo renal transplantation [2]. There are two types of dialysis: hemodialysis (HD) and peritoneal dialysis (PD). The dialysis modality is chosen for every patient considering both medical and nonmedical factors [3]. Hemodialysis should be performed by a healthcare professional. In this type, blood is pumped out of the body to an artificial kidney machine. The machine drains blood, bathes it in a special dialysate solution, removing waste substances and fluid, and after that, returns it to the patient's bloodstream. Hemodialysis is usually performed several times a week and lasts for 3–5 hours. Muscle cramps and hypotension are possible hemodialysis complications [4]. In peritoneal dialysis, a catheter will be placed in the patient's belly and the peritoneum acts as a natural filter [5]. Unlike HD, no specialized in-patient equipment is required and PD can be done at home [6, 7]. Dialysis imposes a high cost on the healthcare system. Based on a comprehensive study, in low and middle-income countries, the annual average cost per patient for HD is about $30,079 and $ 28,592.45 for PD. The cost to society has a wide spectrum, consisted of direct medical hospital, direct nonmedical, and indirect cost [8]. Generally, dialysis patients face several physical, psychological, and social issues that can have lasting effects on their life [2]. One of the unaddressed issues is sexual dysfunction, which is defined as lack of or decrease in sexual desire and difficulty with sexual arousal, pain during intercourse, or orgasmic dysfunction, whether organic or physiologic [9–11]. This is frequently observed in dialysis patients and ranged 41–93%, which poses multifactorial etiology, including hormonal imbalance, vascular and neurogenic dysfunctions, depression, and using antihypertensive (i.e., beta-adrenergic antagonist and diuretics) and antidepressant drugs. [12, 13]. Maintenance dialysis is a life-long treatment, and this is very likely to endanger the occupational, familial, and social status of a dialysis patient [14]. Also, several studies suggest that sexual dysfunction drastically interferes with quality of life (QoL), since any changes in sexual activity affect the person's perception of health. Studies showed that the side effects of the two conventional methods of dialysis are different on a patient's sexual health. Moreover, there is a huge difference between the procedures of HD and PD, and all these factors might have various effects on different aspects of people's personal lives [15]. Therefore, we sought to compare the sexual dysfunction prevalence between ESRD patients, both male and female, undergoing hemodialysis and peritoneal dialysis.

## 2. Materials and Methods

In this cross-sectional analytical study, 190 adults (85 HD and 85 PD subjects) with ESRD being on dialysis who were admitted in (HD patients) or had follow-up at (PD patients) Namazi and Ali-Asghar Hospitals, affiliated to Shiraz University of Medical Sciences, Shiraz, Iran, were included. A subject was included using purposeful convenience sampling if aged 15–65 years, being on dialysis for at least 2 months, and had no any other organ failure. Subjects were excluded if did not undergo dialysis at least twice a week (*n* = 6), were illiterate (*n* = 0), used medications affecting sexual function (*n* = 5), or underwent massive surgeries or other organs' failure (*n* = 9). The data gathering form consisted of demographic variables, underlying the cause of CKD, duration of CKD, dialysis duration and number of sessions per week, selected laboratory markers, comorbidities, surgical history, and drug history. Two widely used sexual function questionnaires were utilized in this study. For male subjects, the erectile function (EF) domain of the International Index of Erectile Function (IIEF) questionnaire was calculated. The Persian version of this questionnaire has been used in hemodialysis patients previously [16]. Also, the Female Sexual Function Index (FSFI) was calculated from the questionnaire [17].

Qualitative and quantitative variables were described using frequency (%) and mean ± standard deviation (SD) (or median (interquartile range (IQR))), respectively. Group comparison was done by Pearson's chi-square test for qualitative variables and the independent *t*-test or Mann–Whitney *U* test if normal distribution was not present. Statistical analysis was carried out using Statistical Package for Social Sciences (SPSS) (Version 16.0. for Windows, Chicago, SPSS Inc). *P* value ≤ 0.05 was considered statistically significant.

## 3. Results

A total of 170 patients (mean age of 49.34 ± 11.7 year; 52.9% female) were eligible for data analysis. Baseline data comparison between HD and PD groups is given in Table 1. Gender, age, BMI, prevalence of diabetes, prevalence of hypertension, marriage duration, and disease duration were not statistically different (*P*=0.035 = 0.999, 0.092, 0.996, 1.000, 0.438, 0.297, and 0.784, respectively). But PD patients had statistically higher rates of smoking history (*P*=0.03) and surgical history (*P*=0.016). In addition, HD patients were on dialysis for a longer time, compared to the PD patients (*P*=0.017).

Laboratory data are given in Table 2, in which they were not significant differences in terms of blood glucose, creatinine, hemoglobin, platelet, cholesterol, and triglyceride levels. Only the BUN level was significantly higher in the PD group (*P*=0.001).

We compared FSFI scores and EF scores of IIEF between HD and PD groups, separately (Table 3). In females (FSFI scores), all scores were higher, better sexual function, in HD group; although, scores reached a significant level just for desire, orgasm, and satisfaction domains, as well as the total score (*P* = 0.03, 0.016, 0.02, and 0.039, respectively). In males (EF scores), erectile function was significantly better in the PD group (*P*=0.035).

## 4. Discussion

Disturbance in sexual function is a serious and prevalent complication among CKD patients which imposes harmful effects on their QoL. Based on previous studies, more than 80% of dialysis patients with chronic renal failure complain of erectile dysfunction [18–20], and a high percent of women treated with dialysis experience impaired sexual function [21]. Erectile dysfunction in men is associated with some diseases [22].

Sexual dysfunction in women unlike men is subjective and more complicated and its treatment remains challenging [23]. To get a more comprehensive view and evaluate sexual dysfunction among CKD patients, we compared sexual function among male and female patients in two groups of HD and PD. The findings of this study showed that among men, erectile function was better in peritoneal groups; however, FSFI's score in women treated with hemodialysis was higher indicating a better sexual function in this group.

Few studies have compared sexual dysfunction between HD and PD patients. Wu et al. [24] reported that after a year, patients on HD had better sexual function than those on PD. Based on a study conducted by Toorians et al. [25], prevalence of sexual dysfunction was 100% in HD and 67% in PD. Also, Guan et al. [26] showed that the prevalence of SD was similar between patients on HD and PD. Moreover, Basok et al. [27] showed that the rates of sexual dysfunction were about 81% and 67% in the PD and HD patients.

Pathophysiology of sexual dysfunction in dialysis patients is complex. Therefore, we need to consider different parameters according to gender, age, underlying conditions (especially diabetes and hypertension as the most common causes of ESRD), as well as personality and psychological status of every patient to choose the suitable method of dialysis. Many studies have proved that this problem, besides anticipated factors, is strongly associated with factors such as stress, depression, and sleep disorders [28–30]. The result of this study showed that the prevalence of sexual dysfunction in PD and HD patients is different between genders pointing to the importance of considering specific dimensions and individual parameters.

Sexual dysfunction is a common problem in the female dialysis population. Dialysis imposes negative effects on different aspects of patients' life and generally decreases their QoL and causes sleep disorder and depression [28]. A great percentage of uremic women complain of decrease in libido and pain during intercourse [31]. Azevedo et al. claimed that the women with impaired sexual function had lower serum creatinine and phosphate level that can show association with nutritional status [32]. Guan et al. reported that the main risk factors for decreased libido and lack of orgasm in CKD female patients are anemia, depression, and using beta-blockers [26].

Studies demonstrated that the increased calcium level is associated with sexual dysfunction in male undergoing PD referring to the vascular etiology of erectile impairment [32]. The reproductive hormonal disturbance is the other reason for erectile dysfunction [16]. In one study, about 34% of patients had low serum testosterone levels [32]. Therefore, controlling these parameters can help patients to improve their sexual functions. The present study had a major limitation as we did not assess the just-mentioned laboratory parameters.

From clinical point of view, the exact role of dialysis on sexual dysfunction is not still clear. First, major underlying conditions of dialysis patients, i.e., hypertension and diabetes, can develop sexual dysfunction through vasculopathy and neuropathy; hence, a synergistic mechanism would be plausible. Second, the mean duration of dialysis was more than 10 years. So, we were not able to track the early effects of dialysis on sexual dysfunction. Third, on the other hand, dialysis can increase appetite (normal BMI in our sample population) and decrease blood urea nitrogen levels, which theoretically might pose a positive effect on sexual dysfunction. By and large, a prospective cohort study on new dialysis patients who are investigated for baseline sexual function would elucidate this question. Besides, diabetes and hypertension pandemics in this millennium are accompanied with the increase of dialysis patients. Therefore, there is an upcoming need to maintain quality of life in this population. A baseline simplified sexual function survey can be part of clinical evaluation for this program.

Apart from being a single center study with a small number of patients, this study was limited by lack of data about ratio of patients with uncontrolled diabetes or anemia, as well as use of supplemental testosterone or hormone replacement therapy (HRT) such as PDE5 inhibitors and estrogen. Of note, psychosocial parameters can elaborate on sexual function. For example, it is shown that economic status can be associated to the presence of sexual dysfunction in noncommunicable diseases [33].

More studies are warranted to evaluate more physiologic, social, and psychologic parameters to gain a better and comprehensive grasp of this phenomenon in dialysis patients. Noticeably, future studies must consider reliable and specialized tools in these patients. For example, the correlation of hbA1c with glycemic control is questioned in both hemodialysis and peritoneal dialysis patients, that is, several adjustments should be considered by physicians to correctly interpret the result of hbA1c [34]. In addition, no sexual dysfunction questionnaire has been specifically designed for dialysis patients. To our knowledge, general questionnaires such as FSFI, SF-36, CHEQ (“CHOICE study” Health Experience Questionnaire including SF-36 and 14 dialysis-specific domains), and IIEF have been utilized by in-line studies, so far [24, 27]. For future research studies as well as clinical purposes, both simplified and extended specialized questionnaires should be constructed.

## 5. Conclusion

Sexual dysfunction is highly prevalent among CKD male and female patients treated with both HD and PD. Dialysis patients suffer from several problems such as diet restrictions, financial problems, sexual dysfunction, worries about their marriage, and change in their responsibilities in their personal life. These problems, especially sexual dysfunction, affect patients' QoL. Considering dialysis as a life-long treatment, this part of a patient's life must be taken seriously by the healthcare system. We should try to choose the most suitable method for patients based on their personality and individual conditions. Healthcare systems should be sensitive to detect this problem in dialysis patients and try to improve the sexual function in patients by choosing effective treatments.

## Figures and Tables

**Table 1:** Comparing baseline data between HD and PD groups.

Variable	HD (*n* = 85)	PD (*n* = 85)	*P*
Gender			
Female	45 (52.9)^1^	45 (52.9)	0.999^3^
Male	40 (47.1)	40 (47.1)	
Age (year)	47.82 ± 12.01^2^	50.85 ± 11.25	0.092^4^
BMI	23.24 ± 3.76	23.24 ± 4.49	0.996^4^
Cigarette			
Current smoker	0 (0)	2 (2.4)	0.03^3^
Ex-smoker	1 (1.2)	7 (8.3)	
Negative	84 (98.8)	75 (89.3)	
Diabetes			
Positive	29 (34.5)	28 (33.3)	1.000^3^
Negative	55 (65.5)	56 (66.7)	
High blood pressure			
Positive	52 (61.2)	46 (54.1)	0.438^3^
Negative	33 (38.8)	39 (45.9)	
Drug history			
Negative	7 (8.6)	9 (11.1)	0.232^3^
Antihypertensive	34 (42)	26 (32.1)	
Antiglycemic	22 (27.2)	17 (21)	
Antidyslipidemia	3 (3.7)	2 (2.5)	
Other medications	15 (18.5)	27 (33.3)	
Surgical history			
Positive	16 (18.8)	31 (36.5)	0.016^3^
Negative	69 (81.2)	54 (63.5)	
Marriage duration (year)	23.61 ± 12.91	25.61 ± 11.87	0.297^4^
Disease duration (year)	20.91 ± 18.31	19.99 ± 24.76	0.784^4^
Dialysis duration (year)	15.57 ± 13.10	11.53 ± 7.32	0.017^4^

^1^Frequency (percentage). ^2^Mean ± standard deviation. ^3^Chi-square test. ^4^Independent *t*-test (Kolmogorov–Smirnov test, *P* > 0.05).

**Table 2:** Comparison of laboratory data between HD and PD groups.

Variable	HD (*n* = 85)	PD (*n* = 85)	*P* ^3^
Blood glucose	102.51 ± 53.15^1^	127.61 ± 135.71	0.129
BUN	21.5 (16.5–39)^2^	33.5 (22–56.75)	0.001^4^
Creatinine	7.33 ± 3.27	6.96 ± 2.54	0.442
Hemoglobin	11.26 ± 3.24	13.79 ± 20.29	0.306
Platelet	166.35 ± 54.6	181.53 ± 59.68	0.117
Cholesterol	142.96 ± 47.63	154.97 ± 54.97	0.163
Triglyceride	133.91 ± 66.61	158.4 ± 82.43	0.057

^1^Mean ± standard deviation. ^2^Median (interquartile range). ^3^Independent *t*-test. ^4^Mann–Whitney *U* test (Kolmogorov–Smirnov test, *P* value = 0.006).

**Table 3:** Comparison of FSFI scores and EF scores of IIEF between HD and PD groups.

	HD (*n* = 45)	PD (*n* = 45)	*P* ^3^
FSFI			
Desire	3.15 ± 1.66^1^	2.43 ± 1.43	0.03
Arousal	2.77 ± 1.92	2.08 ± 1.74	0.076
Lubrication	3.6 (0.3–4.35)^2^	2.4 (0.3–3.6)	0.082^4^
Orgasm	3.23 ± 2.12	2.12 ± 2.14	0.016
Satisfaction	4 (0.2–6)	1.2 (0–4.6)	0.02^4^
Pain	3.29 ± 2.28	2.67 ± 2.32	0.197
Total score	22.2 (3.8–26.75)	14.9 (2–23.25)	0.03^4^

IIEF			
EF	9.02 ± 4.32 1	11.42 ± 5.62	0.035

^1^Mean ± standard deviation. ^2^Median (interquartile range). ^3^Independent *t*-test. ^4^Mann–Whitney *U* test (Kolmogorov–Smirnov test, *P* value = 0.026 lubrication, 0.47 satisfaction, and 0.047 total score).

## Data Availability

The datasets during and/or analyzed during the current study are available from the corresponding author upon request.

## Abbreviations

CKD: Chronic kidney disease
ED: Erectile dysfunction
ESRD: End-stage renal disease
FSFI: Female Sexual Function Index
D: Hemodialysis
IIEF: International Index of Erectile Function
PD: Peritoneal dialysis.
